# Molecular Targets of Brown Algae Phlorotannins for the Therapy of Inflammatory Processes of Various Origins

**DOI:** 10.3390/md20040243

**Published:** 2022-03-30

**Authors:** Natalya N. Besednova, Boris G. Andryukov, Tatyana S. Zaporozhets, Tatyana A. Kuznetsova, Sergey P. Kryzhanovsky, Svetlana P. Ermakova, Irina V. Galkina, Mikhail Yu. Shchelkanov

**Affiliations:** 1 Somov Research Institute of Epidemiology and Microbiology by Federal Service for Surveillance on Consumer Rights Protection and Human Wellbeing, 690087 Vladivostok, Russia; andrukov_bg@mail.ru (B.G.A.); niiem_vl@mail.ru (T.S.Z.); takuznets@mail.ru (T.A.K.); adorob@mail.ru (M.Y.S.); 2 School of Medicine, Far Eastern Federal University (FEFU), 690091 Vladivostok, Russia; galkina.iv@dvfu.ru; 3 Medical Association of the Far Eastern Branch of the Russian Academy of Sciences, 690022 Vladivostok, Russia; priemmodvoran@mail.ru; 4 Elyakov Pacific Institute of Bioorganic Chemistry, Far Eastern Branch of the Russian Academy of Sciences, 690022 Vladivostok, Russia; svetlana_ermakova@hotmail.com; 5 Federal Scientific Center of the East Asia Terrestrial Biodiversity, Far Eastern Branch of the Russian Academy of Sciences, 690091 Vladivostok, Russia; 6 Zhirmunsky National Scientific Center, Marine Biology of the Far Eastern Branch of the Russian Academy of Sciences, 690091 Vladivostok, Russia

**Keywords:** phlorotannins, marine algae, anti-inflammatory effect, antioxidant effect, inflammatory processes, NF-kB signaling pathway, MAPK signaling pathway, Nrf2-Ho-1 signaling pathway, arachidonic acid signaling pathway, JAK-STAT signaling pathway, AP-1 signaling pathway

## Abstract

Inflammatory reactions are part of a complex biological response that plays a vital role in the appearance of various stimuli resulting from tissue and cell damage, the invasion of pathogenic bacteria, and the formation of the subsequent adaptive immune response. The production of many triggers and mediators of inflammation, which are inducers of pro-inflammatory factors, is controlled by numerous differentiation programs, through which inflammation is resolved and tissue homeostasis is restored. However, prolonged inflammatory responses or dysregulation of pro-inflammatory mechanisms can lead to chronic inflammation. Modern advances in biotechnology have made it possible to characterize the anti-inflammatory activity of phlorotannins, polyphenolic compounds from brown seaweed, and the mechanisms by which they modulate the inflammatory response. The purpose of this review is to analyze and summarize the results of numerous experimental in vitro and in vivo studies, illustrating the regulatory mechanisms of these compounds, which have a wide range of biological effects on the body. The results of these studies and the need for further research are discussed.

## 1. Introduction

Inflammation is a typical multi-stage pathological process that develops in response to the action of various damaging factors of an exogenous or endogenous nature, characterized by the development of the body’s protective and adaptive vascular-mesenchymal reactions [1,2,3,4]. These etiological factors can cause acute responses, chronic inflammatory responses, or both in the heart, pancreas, liver, kidneys, lungs, brain, intestinal tract, and reproductive system, potentially leading to tissue damage or disease. Both infectious and non-infectious agents and cell damage activate inflammatory cells and trigger inflammatory signaling pathways, most commonly, NF-Kb (nuclear factor kappa-light-chain-enhancer of activated B cells.), MAPK (mitogen-activated protein kinase) and JAK-STAT (Janus kinases (JAKS)—signal transducer and activator of transcription proteins) [5]. During acute inflammatory responses, cellular and molecular interactions effectively minimize the effects of injury or infection. This mitigation process helps restore tissue homeostasis and resolve inflammation. However, uncontrolled acute inflammation can contribute to chronic inflammation or various diseases—type 2 diabetes, arthritis, cancer, atherosclerosis, cardiovascular diseases, Parkinson’s and Alzheimer’s diseases, etc. [6].

The characteristic clinical signs of inflammation are redness, swelling, fever, pain, and loss of tissue function. These symptoms result from local immune, vascular, and inflammatory cell responses to infection or injury [7]. Inflammatory processes are based on general biological mechanisms that do not depend on the individual characteristics of organisms, localization, or type of stimulus. They involve the coordinated activation of signaling pathways (typically NF-κB, MAPK, and JAK-STAT) that regulate mediator levels in resident tissues and cells [8].

Many anti-inflammatory therapeutic agents are currently available, particularly non-steroidal anti-inflammatory drugs and opioids, but their use is often accompanied by adverse organ and system side effects. In addition, their use has several contraindications. In this regard, at present, much attention is paid to the search for natural anti-inflammatory compounds, including among biologically active substances from marine organisms, polyphenols in particular [9,10]. Among the polyphenols of marine brown algae, phlorotannins attract specific attention, which are potent modulators of biochemical processes associated with inflammation [11,12,13].

The purpose of this review is to assess the current state of research on the anti-inflammatory properties of phlorotannins from marine brown algae and the molecular mechanisms of their implementation.

## 2. General Characteristics of Seaweed Polyphenols

Natural polyphenols are secondary metabolites of terrestrial and aquatic (including marine) plants. Over 10,000 naturally occurring polyphenols have been identified and characterized to date [11]. Marine polyphenols are unique algae metabolites not found in land plants [12,13]. These phenolic compounds are structural components of the algal cell wall and protect them from adverse physical and chemical environmental factors [14,15]. The great interest of the scientific community in the use of natural phenolic compounds from seaweeds is due to the growing knowledge of their biological activity and health benefits. It is no coincidence that marine brown algae, the richest in polyphenols, are widely used as food by the population of Southeast Asia, Japan, and coastal countries of Western Europe [16].

Among brown seaweed phenolic compounds, many phlorotannins (PTs) family members have pronounced biological activities, including algaecidal, antioxidant, anti-inflammatory, antidiabetic, and anticancer activities [13,17,18,19,20].

### 2.1. Classification of Phlorotannins

**Figure 1:**
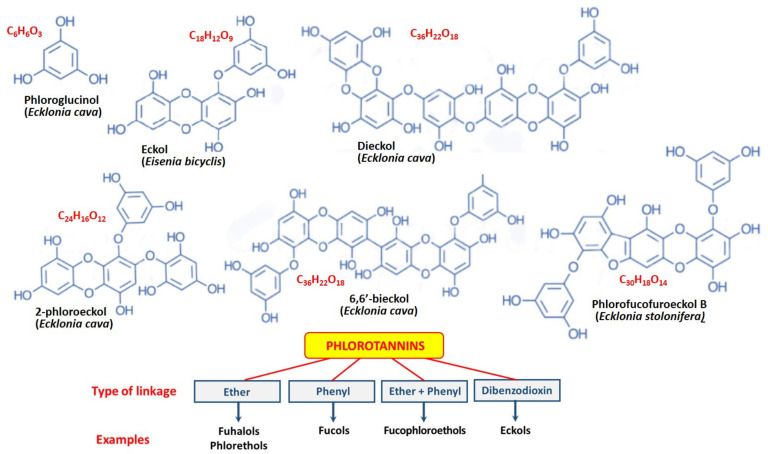
Chemical structure and classification of major groups of phlorotannins (PTs) from seaweeds.

The term “phlorotannins” (PTs) refers to a group of compounds that include dieckols, eckols, dioxinodehydroeckols (eckstolonols), florofucofuroeckol-A, 7-floroeckol, 8,8′-bieckol, fucofuroeckol-A, and 6,6′-bieckol. PTs classification is based on the types of elements between links of phloroglucinol. There are four subclasses with ether linkage (fuhalols and phlorethols), fucols with phenyl linkage, fucophloroethols with simple ether and phenyl linkage, and eckols (dibenzodioxin linkage) [21] (Figure 1).

In seaweed, phloroglucinol is produced as monomeric phloroglucinol, which polymerizes to form simple or giant molecules [22]. According to Heffernan et al. [13], most low molecular weight PTs are represented by 4–16 phloroglucinol monomers. The location of hydroxyl groups in PT is crucial for the manifestation of their anti-inflammatory activity [22,23]. The character of polymerization mediates the formation of various PTs structures, which have many effects, mainly antioxidant, antibacterial, antiviral, antifungal and antiparasitic, antitumor, antidiabetic, etc. In connection with the purpose of the review, let us dwell on the anti-inflammatory effects of these unique compounds.

### 2.2. The Content of PTs in Brown Seaweed

As mentioned above, the composition of the polyphenol fraction of brown algae is characterized by the predominant content of PTs, which are their main cytoplasmic component. These compounds are contained intracellularly in both free and bound states. About 90% of the total PTs are free form in membrane-bound vesicles (phazoids) [7,24,25]. The bound fraction of PTs (more than 10%) resides in the cell wall, where these compounds form a complex with alginic acid and act as structural components of cell membranes, regulating osmotic pressure. According to various data, PTs constitute 13–15% or more of the dry matter weight of algae [26]. Their content may vary depending on the algae type and the geographical growth region [12,24,27]. The highest content of PTs was found in *Eisenia bicyclis* (Japan), *Ecklonia cava, Fucus vesiculosus* (Norway and Russia)*, Fucus serratus, Ascophyllum nodosum* (Norway and Russia), and *Ecklonia kurome*. At the same time, the highest anti-inflammatory activity was observed in PTs obtained from *E. bicyclis, E. cava,* and *F. vesiculosus* [7,12,21,27].

It should be noted that the highest PTs concentration was noted in algae growing in the littoral zone. For comparison, the content of these compounds in green and red algae does not exceed 3.2% [7].

### 2.3. Extraction and Structure PTs

PT extraction efficiency is influenced by extraction time, solvent type, pressure/temperature, solvent-to-solid ratio, and the seaweed’s dry particle size [25]. In addition, the chemical composition of PTs depends on the type of algae and the conditions of their growth of algae, as well as on the mining season, extraction technologies (using organic solvents, microwave-assisted extraction, ultrasound-assisted extraction, enzyme-assisted extraction, pressurized liquid, and supercritical fluid), and several other factors [14,15]. The traditional method of PTs extraction uses organic solvents (ethanol, acetone, methanol, ethyl acetate) followed by purification by chromatographic methods [26,27,28]. Ethyl acetate extraction gives the highest yield of phenols [29]. Supercritical carbon dioxide is also used to extract PTs [30].

For the structural characterization of PTs and the quantitative and qualitative determination of individual components, liquid chromatography-mass spectrometry (LC-MS) followed by UV-detection in the range of 260–280 nm has been used in recent years. In addition, other analytical platforms, such as matrix-assisted laser desorption/ionization time-of-flight mass spectrometry (MALDI-TOF-MS) and NMR spectroscopy coupled with HRMS, are increasingly being used for the same purpose. However, the limiting factor in the choice of the method is the relatively large molecular weight of some oligomers that make up the PTs and the high content of polysaccharides in the algae matrix, making it difficult to isolate and determine the phenol fraction and lack of available commercial standards. Modern analytical methods for isolating and identifying PTs are presented in detail in the reviews [30,31,32,33,34].

The presented characteristics make it possible to conclude the structural and functional diversity of PTs of brown algae [35,36]. To date, about 150 PTs have been isolated from these hydrobionts [15,37], many of which have anti-inflammatory and antioxidant properties. The most studied agents among them are florofucofuroeckol A (PFF-A) and florofucofuroeckol-B (PFF-B), as well as dieckol and 6,6′-bieckol [38,39,40]. However, other PTs, which exhibit high anti-inflammatory activity, are gradually being investigated and proposed as the basis for creating therapeutic agents [40,41].

For example, Phasanasiphon & Kim [39] described low toxicity (90% of cells remain viable) trifuhalol obtained by ethyl acetate extraction from the alga *Agarum cribrosum*. Using gel permeation chromatography, six fractions (F1–F6) were obtained from the extract, of which SF5 and the original extract (EAF) showed intense inhibitory activity in the RAW264.7 macrophage culture against nitric oxide (NO), as well as the expression of iNOS, COX-2, IL-1β, IL-6, and TNFα by RAW264.7 macrophage culture against nitric oxide (NO), as well as the expression of iNOS, COX-2, IL-1β, IL-6, and TNFα [39]. Dose-dependent inhibition of hyaluronidase production by trifuhalol is of great interest. This enzyme depolymerizes hyaluronic acid, which induces pro-inflammatory immune responses. In the future, this property of PT can be used to create a remedy for protection against allergies and inflammation.

To facilitate further identification of these polyphenolic compounds by researchers, Shrestha et al. [41] have created a library of the reported to date phlorotannins. In addition, the proposed database lists the main biological properties of PT with recommendations for the potential use of these unique compounds as food supplements.

### 2.4. Toxicity of Phlorotannins

There are few studies on the relative safety profile of phlorotannins in humans. The latest data highlighting the toxicity of algae PT are summarized in the informative review by Negara et al. [42], in which the authors define these compounds as having low toxicity to cell cultures, invertebrates, macro-and microalgae, plants, animals (fish, mice, rats, and dogs) as well as people. However, mild side effects have been reported in humans, fish, and dogs.

The results of the study on the safety of crude extracts with a high content of phlorotannins and dieckol on volunteers showed no side effects [43,44,45,46]. The daily dose for adults was 230 mg/day for persons under 14 years of age and 263 mg/day for patients over 14 years of age. It is noteworthy that the European Commission on Diet, Nutrition, and Allergy Food Safety Authority (EFSA), following regulation No. 258/97, declared that new food additives based on phlorotannins are safe for human consumption [47].

### 2.5. Bioavailability of Phlorotannins

The bioavailability of biologically active substances is determined by the rate and degree of overcoming metabolic barriers to reach the target site (target), followed by the induction of biological reactions. Therefore, it is a critical factor affecting the biological activity of in vivo compounds. Specific PTs compounds are bioavailable if they reach their target receptor through the circulation in their biologically active form, without any form of degradation or metabolism. The bioavailability of these compounds is assessed by measuring the amount and activity of the compound in the blood serum, the fraction of undegraded PTs in the urine, and its specific metabolites after interaction with a particular receptor [48].

Some studies have shown the metabolism of phlorotannins during their passage through the gastrointestinal tract and absorption into the blood, transportation to organs, and final excretion in the urine [9,18,44,48,49]. The ability of these compounds to act as effective in vivo bioactive molecules depended on the degree of their biotransformation and conjugation during absorption from the gastrointestinal tract to the liver. For example, Corona et al. [9,18] studied high molecular weight PT from the alga *A. nodosum* in an in vitro digestion model and healthy volunteers. They showed that the colonic microbiota fermented these biologically active substances with the formation of low molecular weight metabolites with very little polymerization (for example, hydroxytrifuhalol A, 7-hydroxyeckol, and C-O-C dimer of phloroglucinol), which were found in plasma and excreted in the urine. Bioavailable low molecular weight phlorotannins have been shown in clinical trials [44] to prevent DNA damage, especially in obese people who tend to have significant DNA damage.

## 3. Anti-Inflammatory Effect of Brown Algae Phlorotannins

### 3.1. Models for Testing Anti-Inflammatory Compounds

Many screening studies devoted to the search for anti-inflammatory phlorotannins and their targets in the body in seaweeds have been performed in vitro [50,51,52,53]. These works highlight the anti-inflammatory activity of PTs of various algae species, differences in PTs obtained from the same type of algae, but growing in different geographical regions, the influence of methods for isolating biologically active molecules, extractant, degree of purification, etc.

For the screening of anti-inflammatory compounds in vitro, the RAW 264.7 mouse peritoneal macrophage cell line is most often used, less often, the primary culture of mouse macrophages or other cell cultures are used, and depending on the objectives of the study, *E. coli* lipopolysaccharide (LPS), which induces the production of many inflammatory mediators, is also often used to activate these cells. LPS is a component of the outer membrane of gram-negative bacteria that acts as a prototype endotoxin that appears when gram-negative bacteria are destroyed in the host organism [54].

Although in vitro studies are very relevant and provide essential information, they represent only the first stage of a long process. The results obtained do not always correlate with the performance of these in vivo compounds in a more complex and integrated macroorganism system. Animal experiments and in vivo clinical trials are the studies that contribute most to the proper understanding of the real potential of PTs as future animal and human pharmaceuticals.

The classical model for studying the anti-inflammatory properties of various phenolic compounds in experiments in vivo is the reproduction of carrageenan paw edema in mice or rats. In addition, the reproduction of the inflammatory process in the ear tissue of animals under the action of xylene, arachidonic acid, 12*-O-*Tetradecanoylphorbol-13-acetate, and other substances is often used [12,55], as well as the induction of experimental inflammation of the knee joint of animals.

### 3.2. Molecular Targets of the Anti-Inflammatory Activity of Phlorotannins

When developing new bioactive molecules, the final effect they cause is of great importance, but so is the way in which the compounds stimulate the development and implementation of this effect. Biological agents targeting particular inflammatory molecular pathways are integrated into therapeutic strategies for various diseases. In this case, different cellular elements can act as “targets”: receptors, ion channels, enzymes, genes, etc.

#### 3.2.1. Reactive Oxygen Species

**Figure 2:**
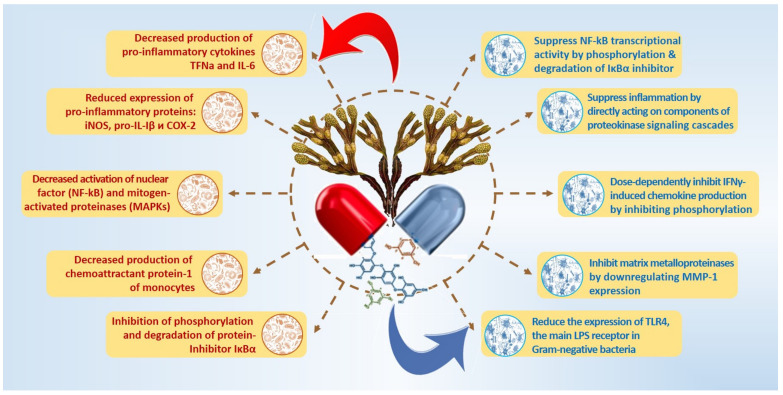
Molecular targets of the anti-inflammatory activity of phlorotannins (PTs).

The generation of activated oxygen-containing and activated nitrogen-containing metabolites—free radical compounds, plays an essential role in cell damage’s pathogenetic mechanism, along with the inflammatory and immune response signaling. This entails modifying the structure of cellular proteins and lipids, disruption of the mechanisms of cell transport and cell division processes, activation of TLR (toll-like receptor) and immune system cells with increased production of pro-inflammatory cytokines, and apoptotic cell death [56]. Reactive oxygen species (ROS), produced during cellular metabolism, are signaling messengers that stimulate inflammatory signaling pathways through protein kinases, transcription factors, and increased genomic expression of pro-inflammatory factors [57]. The onset and development of the inflammatory process are accompanied by oxidative stress [4,58]. The production of pro-inflammatory cytokines largely depends on ROS [59] (Figure 2).

Increased ROS production plays an essential role in the pathogenesis of severe chronic diseases with an inflammatory component, proving common pathophysiological mechanisms between inflammation and oxidative stress [60,61]. The optimal level of ROS in the body is controlled by the antioxidant defense system (AOD) of cells, which includes enzymatic and non-enzymatic units [62].

Natural antioxidants can be protected by natural antioxidants, including plant polyphenols, which strengthen them and restore optimal balance by neutralizing ROS [63,64]. In this regard, modulation of the antioxidant system by antioxidants that inhibit the production of reactive oxygen species and neutralize free radicals can be an integral element of the strategy for preventing and treating diseases with an inflammatory component [64].

Seaweeds’ polyphenolic compounds have strong antioxidant activity [62,65,66,67,68,69,70]. The multifunctional antioxidant activity of PTs is closely related to phenolic rings, which act as electron traps for the absorption of peroxy, superoxide anions, and hydroxyl radicals [65,71,72].

Shibata et al. [73] showed that phenolic hydroxyl groups (OH) and their position (they acted more efficiently in the ortho-position) determine the antiradical activity of PTs. In addition, a positive correlation was observed between antioxidant activity and the number of hydroxyl groups present in the structure of PTs [74].

Algae phlorotannins are more active antioxidants than ascorbic acid, tocopherol, and other known antioxidants [62,73]. For example, the absorption activity of polymerized bifuhalol from the alga *Sargassum ringgoldianum* against superoxide anion radicals is about five times higher than that of catechin, a well-known antioxidant [75].

Antioxidant enzymes—catalases, superoxide dismutases, glutathione peroxidase, and glutathione-S-transferases play a crucial role in maintaining homeostasis in cells. They can also be targets for the anti-inflammatory properties of PTs [76,77]. These compounds can also reduce the level of arginase-2, the increase of which leads to endothelial dysfunction; ref [7,78] concluded that polyphenols have the highest radical scavenging activity at M.m. from 18 to 49 kDa.

#### 3.2.2. NO and NO Synthase

One of the most effective mechanisms of inflammation is the production of nitric oxide (NO) metabolites: nitrites, nitrates, volatile nitrosamines, and the expression of the enzyme—inducible synthase (iNOS). Under an inflammatory stimulus, the iNOS enzyme converts L-arginine to L-citrulline with NO release [70]. NO is a crucial biological messenger in vertebrates, playing an essential regulatory role in various biological processes [70,71,79]. These binary compounds play a vital role in signal transduction from vessels and neurons, bioenergetics, platelet adhesion and aggregation, immunity, and cell death, being the critical biological messenger of vertebrates, participating as a regulator in various biological processes [71]. On the other hand, impaired control of NO level causes pathological conditions such as hypertension, bronchial asthma, cardiovascular dysfunction, arthritis, septic shock, and autoimmune and inflammatory diseases. Picomolar concentrations of NO are physiological, while micromolar concentrations can cause pathological processes [80,81], which depend not only on the concentration but also on the site of its production by NOS isoforms and are distinguished by a variety of manifestations [81].

The pathological effects of NO are caused by an irreversible process, nitrotyrosination of proteins, which leads to the accumulation of modified proteins that contribute to neurodegenerative processes [82]. Therefore, pharmacological regulation of NO synthase activity and NO generation processes for prophylactic and therapeutic purposes is a promising strategy for treating some diseases with an inflammatory component [53].

Rajauria et al. [83] determined that the highest antiradical activity of PTs is observed in M.m. from 8 to 18 kDa. The activity of these compounds decreased with increasing molecular weight. This effect is attributed to forming intra- and intermolecular hydrogen bonds between hydroxyl groups.

Phlorotannins with a more complex structure and high M.m. show a weak anti-inflammatory effect, while low molecular weight PTs can have a strong anti-inflammatory effect. For example, Catarino et al. [84] isolated nine fractions with different M.w. using gel filtration and high PT from an ethyl acetate extract of the brown algae *Fucus vesiculosus*. Fractions with a higher M.m. showed insignificant inhibitory activity against NO, the production of which was induced in the cells of the culture of mouse macrophages RAW264.7 by *E. coli* LPS. At the same time, high concentrations of the studied fractions had a cytotoxic effect on cells. Good results were obtained with fractions having a more straightforward structure and a lower M.m. When fractions F2–F4 and F6 were used, the inhibition of NO production was more than 80%, while the cytotoxic effect of the compounds was absent [84].

It was found that the most significant effect (inhibition of NO production and the expression of iNOS, COX-2, and IL-1β) was obtained when using the low molecular weight fraction F2. This is a result of its ability to inhibit phosphorylation and degradation of the inhibitor κBα (IκBα), which in turn leads to the blocking of the NF-kB signaling pathway. However, this fraction contained minor tetramers of PTs, which, based on MC/MC analysis, were assigned to derivatives of these polyphenols. At the same time, the authors conclude that further studies are needed to prove that the compound found in the F2 fraction is responsible for the intense anti-inflammatory activity. Bogolitsyn et al. [7] concluded that the highest radical scavenging activity is observed in polyphenols at M.m. from 18 to 49 kDa. At the same time, with low molecular weight, PTs have a promising potential for use in functional foods due to their stability and high antioxidant activity [85].

However, there are other opinions as well. For example, Shibata et al. [86] reported higher molecular weight phlorotannins derived from the alga *E. bicyclis* (florofucofuroeckol A, dieckol, and 8,8′-bieckol) had higher hyaluronidase inhibitory activity than low molecular weight ones. Later, similar results were obtained [39] in the study of the inhibitory activity of PT (trifuhalol A) against hyaluronidase from another alga, *Agarum cribrosum*. Thus, the solution to this issue requires accumulating a more extensive array of evidence-based results.

Arginase II is a mitochondrial protein that hydrolyzes L-arginine, thereby reducing NO synthesis, leading to endothelial dysfunction and various cardiovascular diseases. In this regard, this enzyme is recognized as a promising pharmacological target in correcting endothelial dysfunction [78,87]. PTs can reduce the level of arginase-II, the increase of which leads to endothelial dysfunction. In addition, there is information in the literature about the arginase II inhibitory activity of polyphenols from land plants [88,89].

About the protection of the endothelial barrier, there is information characterizing the high potential of PTs as compounds that allow the integrity of the vascular barrier to be maintained.

It is known that changes in the permeability of the endothelial barrier contribute to anaphylaxis, sepsis, acute lung injury, and other diseases [90,91]. Phloroglucinol, eckol, and dieckol from the alga *E. bicyclis* exerted an anti-inflammatory effect in human and mouse umbilical vein endothelial cells in response to the HMGB1 protein. It was found that PT inhibits: (a) LPS-induced release of amphoterin; (b) amphoterin-mediated barrier disruption; (c) expression of cell adhesion molecules; and (d) adhesion/transendothelial migration of leukocytes to human epithelial cells. The results obtained by the authors suggest that phloroglucinol, eckol, and dieckol protect the integrity of the vascular barrier by suppressing hyperpermeability, cell adhesion molecule (CAM) expression, and leukocyte adhesion and migration. Thus, these experiments made it possible to establish that the studied compounds can be the basis for the development of drugs for the treatment of vascular inflammatory diseases [92].

#### 3.2.3. Signaling Pathway Nrf2-Ho-1

Cells protect themselves from oxidative stress by activating antioxidant defense mechanisms. One of the most important regulatory mechanisms is the signaling pathway Keap1-Nrf2-HO-1 [93]. The nuclear transcription factor Nrf2, related to erythroid factor 2, is encoded by the *NEF2L2* gene and regulates the activity of about 250 genes involved in cellular homeostasis [93,94,95,96,97].

The Nrf2 signaling pathway initiates the expression of genes associated with cell defense against antioxidant stress, including antioxidant proteins (e.g., glutamate-cysteine ligases), drug-metabolizing enzymes (e.g., cytochrome c 450), glutathione transferases, molecular chaperones, DNA repair enzymes, and subunits proteasome [98,99].

Under normal physiological conditions, Nrf2 is found in the cytoplasm complex with the regulatory protein Kelch-like ECH-associated protein (Keap1), which determines its low basal expression, which is sufficient to maintain intracellular homeostasis. Under oxidative stress conditions, Keap1 is modified by oxidants and loses the ability to bind Nrf2. As a result, the transcription factor is translocated to the nucleus, forming dimers with Maf proteins. The resulting complexes activate the expression of ARE genes involved in cell defense. Among Nrf2-dependent enzymes, heme oxygenase-1 (HO-1), a powerful anti-inflammatory target, plays a decisive role in gene catabolism [93]. HO-1 expression is induced by oxidative stress by removing its inhibitor Keap1, which promotes its translocation to the nucleus, where the expression of genes involved in the antioxidant response occurs [97]. In animal models, the increase in this expression appears to be protective [100,101].

In recent decades, some substances of natural origin have been proposed to trigger the overexpression of HO-1 in animal models (polyphenols and first compounds containing OH groups in the ortho and para positions). An example is the compounds from terrestrial (quercetin and epigallocatechin, etc.) and water (eckol, phloroglucinol, etc.) sources [98,102,103].

PTs enhance the expression of Nrf2 and HO-1 proteins, reducing the intensity of oxidative stress and inflammation. For example, Kim et al. [103] showed that eckol (*Ecklonia cava*) induced HO-1 expression at the mRNA and protein levels in lung fibroblast cells and protected them from oxidative stress-induced DNA damage and apoptosis by activating the Nfr2-HO-1 signaling pathway. Evidence for the involvement of HO-1 in this process is the inhibition of HO-1 function by zinc protoporphyrin (HO-1 inhibitor), which weakened the protective effect of phloroglucinol against H_2_O_2_. In addition, Rajan et al. [15] showed the biological impact of Diecol against ovarian, liver, and colon cancer in animal models through the activation of the Nfr2-MAPK signaling pathway in liver cells.

Eckol-mediated (from *E. stolonifera*) enhancement of HO-1 expression in HepG2 cells, regulated by activation of the Nrf2 signaling pathway through JNK and PI3K/Act, confirms the possibility of using this PT as a natural antioxidant and cytoprotector [102]. Thus, therapeutic modulation of the Nfr2-HO-1 signaling pathway by brown algae phlorotannins may be a new strategy for preventing and treating diseases with an inflammatory component.

#### 3.2.4. TLR-Signaling Pathway

The transmission of signals from receptors to the genome is one of the most relevant and intensively developing cell and molecular biology areas. TLR-signaling pathways are potential targets for developing new strategies for the treatment of various diseases by precisely targeting specific proteins. Particular attention is paid to information about substances that provide or block (partially or completely) signal transmission in the cell; they recognize bacterial lipopolysaccharides and endogenous molecules that are produced during pathological processes in different tissues, activating the corresponding responses of innate cellular immunity [103,104].

As a rule, TLRs are localized on the cell membrane, act as primary sensors, and exacerbate inflammatory reactions of infectious and non-infectious genesis, translating inflammation into a chronic form [104]. Recognition of bacterial and non-bacterial ligands of pathogen-associated molecular patterns (PAMPs) by specific TLRs leads to the activation of transcription factors such as nuclear factor-kB (NF-kB) and members of the IRF (interferon regulatory factor) family [105]. In addition, ligand binding causes TLR homodimerization or heterodimerization and recruitment of adapter molecules MyD88 (myeloid differentiation primary response gene), TIRAP, TRIF, and TIRP, which cause differentiated excitation of intracellular signaling cascades [106]. Depending on the adapter molecules involved, TLR signaling pathways are subdivided into MyD88-dependent and MyD88-independent pathways [105,106].

Upon excitation of the MyD88-dependent pathway, adapter molecules form a molecular complex with the TIR (toll-interleukin-1 receptor) domain of TLR, thereby triggering the signaling cascade. Excitation of the MyD88-independent pathway is due to the recruitment of other adapter molecules TRIF/TICAM-1 and TIRP/TRAM/TICAM-2 (TIR domain-containing adaptor inducing interferon-beta/TIR domain-containing adaptor molecule 1), which interact with the TIR domain of TLR [104,105,106].

The intracellular activation signal is mediated by the induction of c-Jun N-terminal kinase (JNK), extracellular signal-regulated kinase1/2(ERK1/2), or phosphoinositide-3 kinase [107]. At the final stage, all three pathways ensure the translocation of NF-kB to the cell nucleus. Cell surface TLRs include TLR1, TLR2, TLR4, TLR5, TLR6, and TLR10, while intracellular TLRs are located in the endosome and include TLR3, TLR7, TLR8, TLR9, TLR11, TLR12, and TLR13 [104].

A promising target for developing anti-inflammatory drugs is TLR4, the primary lipopolysaccharide receptor in Gram-negative bacteria. TLR4 also recognizes other ligands, such as heat shock proteins Hsp60 and Hsp70, fibronectin, hyaluronic acid, heparan sulfate, and β-defensin-2 [108,109]. Signal transduction from TLR4 depends on where it is located in the cell. After binding to the ligand, the receptor localized in the cell membrane transmits a signal along the TIRAP-MyD88 (domain-containing adapter protein) pathway. Shortly after that, it is internalized within endosomes but remains active and transmits a call via the TRAM-TRIF pathway [110,111]. It is known that the inhibition of TLR4 expression reduces inflammation and pain intensity [112].

Many authors [4,113] have reported decreased TLR4 expression by polyphenols from terrestrial sources in metabolic syndrome. For example, phlorotannins from *E. cava* (dieckol—DK, 2,7-phloroglucin-6,7-bieckol—PHB, pyrogallol-phloroglucin-6,6-bieckol, and phlorofucofuroeckol A—PFF-A) weaken resistance to leptin, which plays a significant role in the development of obesity. The expression of TLR4 and NF-kB induced by palmitate was most effectively reduced by pyrogallol-phloroglucin-6,6-bieckol at a 1.8 μg/mL concentration in both hypothalamic neurons and microglia, while leptin resistance was inhibited. In terms of efficiency, the studied PTs were arranged as follows: PPB > DK > PHB = PFF-A [114].

#### 3.2.5. NF-kB-Signaling Pathway

The transcription factor NF-kB is a family of inducible transcription factors that regulate an extensive array of genes involved in various immune and inflammatory processes. This family consists of five structurally related members, including NF-kB (also called p50), NF-kB2 (called p52), RelA (called p65), RelB, and c-Rel, which mediate the transcription of target genes by binding to a specific element. DNA, kB enhancer, in the form of various hetero- or homodimers [62]. NF-kB activity is regulated by different mechanisms depending on IkB accumulation and degradation, NF-kB phosphorylation, IKK hyperphosphorylation, and processing of NF-kB precursors [115].

NF-kB plays a crucial role in DNA transcription, cytokine production, and cell survival; it controls immune responses, inflammation, stress, proliferation, and apoptotic cell responses to multiple stimuli [116,117,118]. The study of the activation of NF-kB, the central mediator of pro-inflammatory gene induction, in various models of inflammation provided an opportunity to prove its role in mediating inflammation through the increase in COX-2 and iNOS, followed by the production of PGE2 and NO. The participation of NF-kB in the transcription of the essential pro-inflammatory genes, such as IL-1, IL-6, IL-8, COX-2, iNOS, chemokines, including the most potent monocyte chemoattractant MCP-1, corresponds to the critical role of this factor in the inflammatory regulation response [118].

**Figure 3:**
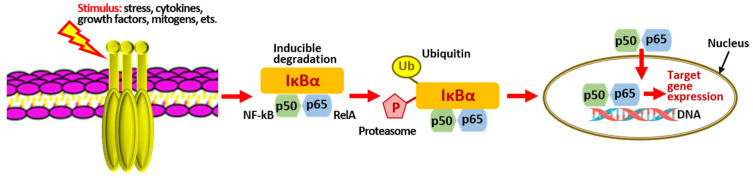
Activation mechanisms of transcription factor NF-kB in inflammation: Inducible degradation of IκBα by IκB (IKK). IKK can be activated by various stimuli, including cytokines, growth factors, mitogens, microbial components, and stress agents. IKK phosphorylates IκBα, triggers ubiquitin-dependent degradation of IκBα in the proteasome, leading to rapid and transient translocation into the nucleus of canonical members of NF-κB.

The PI3K/Act/IKK/NF-kB signaling cascade, in turn, controls NF-kB activation. Prostaglandins, such as PGE2 and leukotrienes, stimulate the transcriptional activity of NF-kB, not through the regulation of IKK but by using alternative mechanisms [119]. There are two distinct pathways for NF-kB activation: classical and alternative [120]. In both cases, NF-kB is released from its inhibitor IkB, which leads to translocation to the nucleus and expression of target genes [58] (Figure 3 and Table 1).

NF-kB interacts with a variety of other signaling molecules and pathways. Crosstalk nodes are mediated by other transcription factors, for example, STAT3 and p53 or the ETS-associated ERG gene [120]. These transcription factors interact directly with NF-kB subunits or affect NF-kB target genes. Cross-links can also occur through various kinases (GSK3-β or RI3K) that modulate the transcriptional activity of NF-kB or affect upstream signaling pathways. Reactive oxygen species and mRNA can also serve as cross-interaction nodes [120,133].

Under physiological conditions, NF-kB is found in the cytoplasm of cells in an inactive form that does not bind to DNA, is a self-regulating system, and is associated with inhibitory κB (IκB) proteins that exist in the α, β, γ, and ε forms. When stimulated by viruses, lipopolysaccharides, mitogens, etc., the intracellular signaling cascade triggers the phosphorylation of IκBα, which is further ubiquitinated and degraded by the proteasome, releasing NF-kB. The latter then moves to the cell nucleus, binds to specific DNA recognition sites, and induces the transcription of pro-inflammatory genes (over 500) encoding biochemical mediators involved in the inflammatory process [119]. As a rule, the best-known p50/p65 and p50/c-Rel dimers are activated via this pathway.

The leading role of NF-kB in the development of inflammation makes it a promising pharmaceutical target for the development of drugs against diseases with a vital inflammatory component. The ability of seaweed phlorotannins to inhibit the transcription factor NF-kB is one of the promising approaches to explain the mechanisms of the anti-inflammatory action of these compounds.

Kim et al. [133] found that the addition of an ethanolic extract of *E. cava* (FT 67%, main component dieckol) to LPS-stimulated macrophages RAW264.7 dose-dependently reduces the levels of TNFα, IL-1β, IL-6, iNOS, COX-2, NO, and PGE2 by inhibiting NF-kB translocation to the nucleus and binding it to the cell’s DNA. Furthermore, in LPS-stimulated BV2 microglial cells, the ability of this extract to inhibit translocation was associated with both IκB degradation and suppression of the mitogen-activated protein kinase (MAPK) pathway, which also contributed to the regulation of pro-inflammatory cytokine biosynthesis [134].

Inactivation of LPS-induced NF-kB transcriptional activity in Diecol-treated RAW264.7 macrophages results from the inhibition of phosphorylation of the p65 NF-kB subunit and its upstream kinases PI3K, Akt, IKKα/β, and IκBα [121]. This inhibition pathway is similar to that previously described for PFF-A in LPS-stimulated RAW 264.7 cells [121,133,134]. The authors found that treatment of LPS-stimulated RAW264.7 macrophages with florofucofuroecol A (20 µM) significantly reduced iNOS and COX-2 mRNA expression. At the same time, the levels of pro-inflammatory cytokines were significantly reduced. The studies’ results indicate that florofucofuroecol A regulates the expression of iNOS and COX-2 through NF-kB-dependent transcriptional control associated with the inhibition of multiple signaling proteins. Similar results are described by Kim et al. [122] for 6,6′-bieckol, which can reduce, depending on the dose, the expression of iNOS, COX-2, and pro-inflammatory cytokines in LPS-stimulated RAW 264.7 cells and BV2 microglial culture cells through the inhibition of the IκB-α/NF-kB and JNK pathways/ p38 MAPK/Akt, in connection with which this PTs can become the basis of a therapeutic agent for the treatment of neuroinflammatory diseases in the future [122].

A recent study by Catarino et al. [123] also showed the inhibition of the NF-kB signaling pathway by phlorotannins from the brown alga *F. vesiculosus*. As described previously, phlorotannin-containing fractions (9 fractions) were obtained [123]. Of all the fractions studied, F2 was the only one that reduced the expression in RAW264.7 macrophages of the three pro-inflammatory proteins tested, iNOS, pro-IL-1β, and COX-2, mediated by several transcription factors, the most important of which is NF-kB. The relationship between iNOS expression and NF-kB transcriptional activity in the presence of LPS has been established previously. [124].

Thus, the pronounced suppression of iNOS by PTs indirectly confirmed the involvement of NF-kB in this process. The authors tested these samples of phlorotannins for the ability to inhibit the phosphorylation and degradation of the IκBα inhibitor and the translocation of NF-kB to the nucleus. It was found that the presence of F2 completely abolished the effects of LPS, maintaining the levels of phosphorylated and total IκBα as in the control sample (without the action of LPS). Since IκBα is responsible for maintaining isolated and inactive NF-kB in the cell cytoplasm, the inhibition of its phosphorylation and degradation indirectly indicates that NF-kB translocation was blocked and transcriptional activity was suppressed. Interestingly, neither of the other two samples tested could prevent the decline in IκBα levels, which meant that the protein was still being cleaved by the proteasome, and NF-kB continued to move into the nucleus. Thus, the *F. vesiculosus* alga is a source of PTs capable of suppressing the transcriptional activity of NF-kB by inhibiting phosphorylation and degradation of the IκBα inhibitor [118,125,126,133].

In addition to AKT/IkB-mediated NF-kB inactivation, dieckol, derived from the brown alga *E. cava*, has been shown to reduce phosphorylation of p38 and ERK (signal-regulated kinases) in three different human liver cell lines. LX-2 and HSC-T6 (liver stellate cells), as well as HepG2 (hepatocellular carcinoma cells) [94,127,128,129,130,131,132,133]. PTs reduced the expression of α-SMA (anti-smooth muscle antibodies) and TGF-1β, increased the sub-G1 phase population, and induced caspase three activation and PARP [poly(ADP-ribose) polymerase] cleavage in HSCs (hematopoietic stem cells). Thus, dieckol suppressed the development of liver fibrosis by activating caspases and JNK activation mediated by miR 134 and the inhibition of NF-kB [39].

Fucofuroeckol A (FF-A), another PT from brown algae *Eisenia bicyclis*, acted on RAW 264.7 cells via the NF-kB and MAPK signaling pathways. In a culture of mouse macrophages, FF-A reduced the hyperproduction of NO and PGE2 induced by LPS and reduced the expression of mRNA and protein iNOS and COX-2, reduced the production of pro-inflammatory cytokines (TNFα, IL-6), monocyte chemoattractant protein-1, and activation of nuclear factor KB (NF-kB) and mitogen-activated protein kinases (MAPKs) [39,84,135,136].

The authors showed that the addition of PFF-A to the RAW264.7 cell culture reduced the phosphorylation of JNK and p38 kinase, but not ERK1/2, stimulated by LPS, indicating that JNK and p38, but not ERK1/2, are involved in the inhibitory effect of PT on LPS-induced iNOS and COX-2 expression and NF-kB activation [137].

Yang et al. [127] investigated the mechanisms of the anti-inflammatory action of PT PFF-A from the brown alga *E. cava* in an experiment on mice with experimental radiation dermatitis, one of the most common undesirable side effects in radiation therapy. PFF-A significantly reduced the intensity of clinical symptoms of radiation dermatitis. The decrease in the intensity of acute inflammation in the irradiated skin of mice was accompanied by a decrease in the radiation-induced activation of NF-kB and inflammasomes; the topical application of PT can relieve the symptoms of radiation dermatitis by promoting the healing process. Under the action of the compound, the activation of the Nfr2-HO-1 signaling pathway was also enhanced, which indicated an improvement in the antioxidant status of the animals. At the same time, the expression of Nrf-2-HO-1 in animals treated with PTs was higher on day 14 and not on day 21. The authors believe that the Nrf2 signaling pathway alleviates the symptoms of radiation dermatitis and NLRP3 inflammasome activity, mainly in the early acute phase of the process. However, even on day 21, the expression levels of Nrf2-HO-1 in mice treated with PTs remained higher than in animals that did not receive it [127].

Crosstalk occurs between the NF-kB and Nfr2 pathways: Nfr2 signaling inhibits the NF-kB pathway and vice versa [128,129]. Through the NF-kB and Nfr2/HO-1 signaling pathways, an extract of the alga *E. cava* with a high content of PT dieckol also showed an anti-inflammatory effect [128,130]. In a mouse model of septic shock and LPS-induced hyperinflammatory response, a decrease in the level of iNOS, COX-2, TNFα, IL-6, and amphoterin (HMGB-1) in RAW264.7 macrophages treated with the extract was found. In addition, the extract inhibited the NIK/TAK1/IKK/IkB/NF-kB pathway and increased Nfr2 and HO-1 expression [130].

In addition to NF-kB, many other signaling pathways are involved in the pathogenesis of chronic neurodegenerative diseases [128,131,132], which can become targets for algal phlorotannins. Furthermore, some studies have presented the mechanisms of the neuroprotective action of these compounds, confirming that they can be considered candidates for nutraceuticals and as a future drug for the treatment of neuroinflammation in neurodegenerative diseases [39,135,136].

Thus, the NF-kB signaling system is a potential target for creating innovative targeted drugs based on brown algae phlorotannins.

#### 3.2.6. MAPK Signaling Pathway

**Figure 4:**
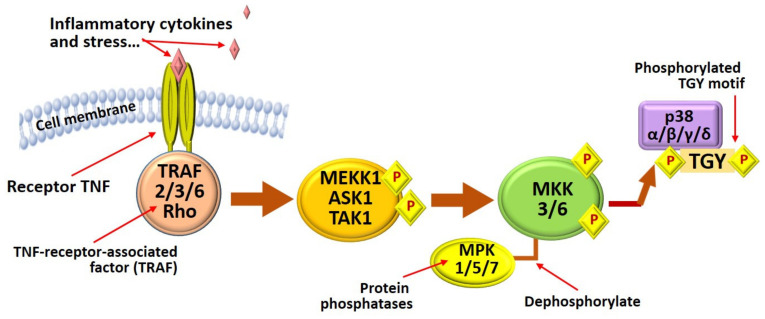
Activation mechanisms of transcription factor MAPK in inflammation. Infectious agents, damaged tissues, or toxins activate membrane Toll-like receptors on immune cells. Receptor ligation stimulates the formation of the TRAF signaling complex with regulatory cellular proteins (Rho). This complex activates TGFβ kinase (TAK1), apoptosis signal-regulating kinase 1 (ASK1), and MAPK/ERK kinase kinase (MEKK1). MAPK signaling induces the expression, activation, and activation of dual specificity phosphatases (DUSPs) that dephosphorylate and inactivate MAPK.

MAPK (mitogen-activated protein kinases) belong to the serine-threonine protein kinases class, activated in response to numerous external influences, including cytokines, growth factors, and matrix proteins, and transmitted signals from the cell surface to the cell nucleus. There are three major mitogen-activated protein kinases (MAPKs): p38 kinase, c-Jun N-terminal kinase (JNK), and signal-regulatory extracellular kinase (ERK). All of them are involved in various cellular signaling pathways that regulate cell growth, proliferation, differentiation, and apoptosis [21,27,137,138,139] (Figure 4 and Table 2).

During activation, MAPKs undergo phosphorylation [37,94,140] and transmit an activating signal further down the chain by the phosphorylation of several target transcription factors, including Nrf2, NF-kB, and AP-1. Among them, p38 MAPK is activated by several pro-inflammatory stimuli such as oxidative stress, UVB, and inflammatory cytokines. In addition, activator protein-1 (AP-1), a transcription factor of the MAPK pathway, can interact with specific DNA sequences called AP-1 sites. AR-1 regulates many cellular processes, including inflammation, proliferation, differentiation, and apoptosis [62,124,141,142], and can be activated by various extracellular stimuli [37,138]. In addition, MAPKs cross-react with other pathways, such as NF-kB, thereby complicating the MAPK signaling pathway and its interactions [140,141,143,144,145]. 

The use of stimulants and inhibitors of MAPK mechanisms is a new promising direction in treating diseases whose pathogenesis is associated with impaired cell differentiation, proliferation, excessive production of cytokines, and regulation of connective tissue growth. Several pharmacological preparations have been developed that directly affect the components of protein kinase signaling cascades. These include inhibitors of PI3K, Act, mTOR, BRAF, B-RAF (V600E), MEK, and ERK, as well as protein kinase inhibitors with a broad spectrum of action [146,147]. Some of these drugs are still undergoing clinical trials, and some are already being introduced into clinical practice.

The ability of phlorotannins to block MAPK pathways provides these compounds with therapeutic potential for protection against inflammation. For example, Phasanasiphon et al. [39] found that the inhibitory effect of trifuhalol, PT from the alga *Agarum cribrosum*, on the production of hyaluronic acid, nitric oxide NO, expression of iNOS, COX-2, IL-1β, IL-6, and TNFα is associated with the effect of this compound on pro-inflammatory signaling pathways, including MAPK. Activation of nuclear factor NF-kB and MAPKs was attenuated by inhibiting NF-kB p65, c-Jun N-terminal kinase, extracellular signal-regulated kinase ½, and p38 MAPK phosphorylation. Previously [148], it was shown that PTs derivatives from the alga *E. cava* [dieckol and 1-(3′, 5′-dihydroxyphenoxy)-7-(2″, 4″, 6″-trihydroxyphenoxy)2,4,9-trihydroxydibenzo-1,4-dioxin] inhibited gene mRNA levels and protein expression of MMP-1, MMP-3 and MMP-13, and iNOS and COX-2, and reduced inflammation via the MAPK signaling pathway.

As is known, particulate matter in the air increases the risks to human health in various diseases: respiratory [149], cardiovascular [150], and pneumonia [151]. Fine particles with a diameter of less than 2.5 mm (PM2.5) can penetrate deep into the skin and respiratory tract [152] and induce apoptosis associated with ROS formation. Skin damage caused by PM2.5 manifests itself in inflammatory diseases (atopic dermatitis, acne, psoriasis, aging, and oncology) with the participation of multiple signaling pathways [125]. Experimentally, particulate matter induced endoplasmic reticulum stress, mitochondrial swelling, autophagy, and apoptosis in HaCaT cells and mouse skin tissue [153].

Zhen et al. [154] found that PM2.5 particles increase pro-apoptotic protein levels and activate the MAPK signaling pathway. As eckol has a multi protective effect against several cell lines, including lung fibroblasts [155], human skin fibroblasts [156], Chang liver cells [152], and human skin keratinocytes [153], it was used in an experiment to protect HaCaT keratinocytes from apoptosis induced by PM2.5. Under the influence of PM2.5, the level of Bax (a pro-apoptotic protein) and activated caspase-3 significantly increased, while under the influence of eckol, this indicator decreased. Eckol inactivated proteins associated with the MAPK signaling pathway in keratinocytes (ERK, p38, and JNK) are significantly upregulated by MP2.5. In addition, these PTs protected cells by reducing ROS generation while maintaining mitochondrial stability. Inhibition of the MAPK signaling pathway has been confirmed by using MAPK inhibitors (UO126, SB203580, SP600125) [154].

#### 3.2.7. Arachidonic Acid Signaling Pathway

The arachidonic acid (AA) signaling pathway plays a crucial role in the biology of the cardiovascular system, carcinogenesis, and many inflammatory diseases such as asthma, arthritis, etc., in response to an inflammatory stimulus. Esterified AA, the primary polyunsaturated fatty acid of the phospholipid layer of cell membranes, is hydrolyzed in accessible form by phospholipase A2 (PLA2), which, in turn, is additionally released with the participation of cyclooxygenases, lipoxygenases, and epoxygenases and is metabolized with the formation of pro-inflammatory eicosanoids: prostanoids (prostaglandins—PGD2, PGE2, PG12, prostacyclin PGD1, thromboxane A2), leukotrienes, eoxins, hepaxilins, epoxyeicosatrienoic acids, lipoxins, resolvins, and protectins [148].

Several studies have reported modulating the AA PTs signaling pathway associated with the inhibition of the cyclooxygenase, lipoxygenase, and epoxygenase metabolic pathways. Early work by Shibata et al. [157] found that purified PT from the brown alga *E. bicyclis* can take part in AA metabolism and inhibit a number of its metabolites while exerting an anti-inflammatory effect. In vitro experiments have shown that these phlorotannins inhibit the activity of secretory phospholipases A2 (sPLA2), lipoxygenases, and COX-2. This effect was more pronounced than the well-known LOX inhibitors, resveratrol, and epigallocatechin gallate [158]. The most potent inhibitors of LOX and 5-LOX with values of 38 and 24 μM were dieckol and eckol, respectively. The inhibitory effect of dieckol on COX-1 was 74.7%, eckol on COX-2 was 43% at a dose of FT of 100 μM.

In acute myocardial infarction, lipoxygenases are of particular importance. This family of enzymes is essential for the biosynthesis of leukotrienes from AA and is one of the main factors in the progression of inflammation. Leukotrienes have a pronounced coronary constrictor, proaggregant, arrhythmogenic, and chemoattractant action and promote the formation of free radicals and the accumulation of polymorphonuclear neutrophils—cells with significant prooxidant, lipoxygenase, and proteolytic potential. Given these facts, it can be argued that the anti-LOX and antileukotriene properties of various compounds, the action associated with the arachidonic acid pathway, underlie the development of new approaches to the treatment of cardiovascular diseases [158,159]. Kurihara et al. [159] studied the LOX inhibitor from the brown alga *Colpomenia bullosa*, fucofloretol C, which inhibited soybean LOX with a Ki value of 137 μM.

Biological agents that target highly specific inflammatory molecular pathways have been shown to work in experimental models related to allergies. Potential targets for developing new therapies for allergic diseases include IgE, TH2 lymphocytes and TH2-derived cytokines, IL-4 and IL-5, and activated mast cells releasing bioactive allergy mediators (histamine, tumor necrosis factor, prostaglandin D2, etc.) [158,159,160].

An informative review by Sugiura et al. [12] summarized the materials characterizing the antiallergic effect of PTs from seaweed. Finally, we refer readers to this paper, which outlines an analysis of 99 publications, concluding that phlorotannins alleviate allergic inflammation, acute type I allergic reactions, and chronic type IV allergic reactions through attenuation of the arachidonic acid cascade, regulation of the MAPK/NF-κB signaling pathway, and immunomodulation by inhibiting the activity of inflammation-related enzymes such as phospholipase A2, COX-2, lipoxygenase, and hyaluronidase.

#### 3.2.8. JAK-STAT Signaling Pathway

The JAK/STAT signaling pathway (Janus kinase transducer/transcriptional activator) is a relatively simple and conserved cascade of signal transduction reactions in multicellular organisms. It is regarded as one of the central communication nodes in cell functioning. More than 50 cytokines and growth factors have been identified in JAK/STAT, including hormones, interferons, interleukins, and colony-stimulating factors. JAK/STAT mediated events include hematopoiesis, immunopoiesis, tissue repair, inflammation, apoptosis, and adipogenesis [161,162,163].

After ligand binding, receptors dimerize and activate JAK molecules associated with them, which phosphorylate STAT proteins, creating conditions for their translocation to the nucleus to regulate specific genes [163,164]. Impaired JAK/STAT signaling can lead to various diseases: skin diseases, cancer, rheumatoid arthritis, diabetes, and diseases of the cardiovascular system [162,165]. Inhibition of the JAK/STAT pathway is currently regarded as a promising strategy for treating various diseases, and many potential inhibitors of this pathway are being investigated in the clinic and experiments [165].

PTs from terrestrial and marine sources have shown promising effects on JAK/STAT in inflammatory, oncological, and cardiovascular diseases. For example, curcumin has proven its effectiveness in experiments on cellular and animal models of various diseases [166]. In addition, Kang et al. [167] found that *E. cava* dieckol at doses of 5 and 10 μM was able to dose-dependently inhibit IFNγ-induced production of the MDC/CCL22 chemokine by suppressing phosphorylation and nuclear translocation of STAT1.

Thus, brown algae phlorotannins regulate the synthesis of pro-inflammatory cytokines and gene expression. They inactivate the nuclear factor NF-kB, modulate the mitogen-activated protein kinase (MAPK), JAK-STAT, arachidonic acid, and AP-1 pathways, as well as the Nrf2/Ho-1 signaling pathway, which plays a crucial role in the protection against oxidative stress and inflammation, inhibits phosphatidylinositide-3-kinases, and suppresses the expression of TLR. Their antioxidant activity and ability to inhibit enzymes involved in the production of eicosanoids also contribute to their anti-inflammatory properties. PTs inhibit phospholipase A2, COX, and LOX, which leads to a decrease in the production of prostaglandins and leukotrienes and, as a result, to a decrease in the intensity of the inflammatory process. Due to their ability to modulate various inflammatory signaling pathways, phlorotannins can be considered adequate protection of different cell types from inflammatory damage.

#### 3.2.9. Matrix Metalloproteinases (MMP)

The system of matrix metalloproteinases (MMP) and tissue inhibitors of metalloproteinases (TIMP) are involved in the regulation and degradation of the extracellular matrix in chronic heart failure, arthritis, metastasis of malignant neoplasms, skin aging, and other diseases associated with chronic inflammation [62,156,168,169].

MMPs are produced by resident cells and those involved in inflammatory processes and can destroy almost all macromolecules present in the extracellular matrix [170,171,172,173]. Transcription factors that regulate the production of MMP mRNA are AP-1, AP-2, HIF, PEA-3, and ER. They, in turn, are the final links of the Ras-MAPK/ERK, JAK-STAT, and TGF-β/SMAD signaling pathways [171]. The functions of metalloproteinases are diverse, and the imbalance of their activity can be one of the etiological factors of various diseases, [172,173], therefore, MMP suppression is a promising therapeutic strategy for treating diseases in which chronic systemic inflammation plays an essential role in pathogenesis. Many of the synthetic inhibitors of matrix metalloproteinases have not justified expectations in clinical trials, and, therefore, natural sources are of great importance. Accumulating evidence suggests that various antioxidants (quercetin, β-carotene, epigallocatechin-3-gallate, vitamin E, etc.) suppress ROS-induced MMP expression [174]. BAS from marine sources also exhibit inhibitory activity against MMP [62,156,168], particularly seaweeds. Thus, Liu et al. [62] showed that the expression of MMP-2 and MMP-9 was suppressed by fucofuroecol A and ecol in the human fibrosarcoma cell line HT1080 by blocking the transcription of NF-kB and AP-1.

Joe et al. [156] described the ability of eckol and dieckol from the alga *E. stolonifera* to reduce the expression of MMP-1 and the level of reporter genes of the NF-kB and AP-1 signaling pathways. Suppression of MMP expression in HT1080 cells may be associated with suppression of the NF-kB signaling pathway [166].

#### 3.2.10. Other Targets of Seaweed Phlorotannins

It is known that changes in the permeability of the endothelial barrier contribute to anaphylaxis, sepsis, acute lung injury, and other diseases [90,91,92,175,176,177,178]. Phloroglucinol, eckol, and dieckol from the alga *E. bicyclis* had an anti-inflammatory effect in endothelial cells of the human and mouse umbilical vein in response to the HMGB1 protein (amphoterin is a cytokine mediator, a protein from the group of non-histone HMG proteins secreted by monocytes and macrophages). It was found that PTs inhibits: (a) LPS-induced release of HMGB1; (b) HMGB1-mediated barrier disruption; (c) expression of cell adhesion molecules; and (d) adhesion/transendothelial migration of leukocytes to human epithelial cells. The results obtained by the authors suggest that phloroglucinol, eckol, and dieckol protect the integrity of the vascular barrier by suppressing hyperpermeability, cell adhesion molecule (CAM) expression, and leukocyte adhesion and migration. Thus, these experiments made it possible to establish that the studied compounds can be the basis for the development of drugs for the treatment of vascular inflammatory diseases [92].

## 4. Comparison of the Effectiveness of PTs and Known Anti-Inflammatory Drugs

The use of various experimental in vivo models allowed us to conduct comparative studies of the effectiveness of various phlorotannins and officinal drugs. Abdelhamid et al. [175] isolated fractions with a high content of polyphenols: 2-[2-(3,5-dihydroxyphenoxy)-3,5-dihydroxyphenoxy]-1,3,5-benzenetriol (1); 2,2′-[[2-(3, 5 dihydroxyphenoxy)-5-hydroxy-1,3-phenylene]bis(oxy)]bis(1,3,5-benzenetriol) (2); and 2-[2-[4-[2-(3,5-dihydroxyphenoxy)-3,5-dihydroxyphenoxy]-3,5-dihydroxyphenoxy]-3,5-dihydroxyphenoxy]-1,3,5-benzenetriol (3) from extracts of three species of brown algae—*Cystoseira sedoides* (PHT-SED), *Cladostephus spongiosis* (PHT-CLAD), and *Padina pavonica* (PHT-PAD). Good results have been obtained in mouse ear edema and rat paw edema models using the PHT-SED fraction 30 min prior to xylene application. The percentage of inhibition of ear edema and paw edema was 82.55% and 81.08%, respectively. The same fraction had the highest antioxidant potential, comparable to quercetin and vitamin C (standard antioxidants). Quite good results of the study of anti-inflammatory action were obtained using the PHT-CLAD fraction on the model of ear edema (inhibition was 68.6%). The effect of the reference drug (dexamethasone) was 53.49%, i.e., it was lower than that of the studied fractions [175]. 

Subplantar administration of carrageenan to rats led to edema within 1 h with a maximum amount after 3 h. The anti-inflammatory drug diclofenac reduced paw edema after 3 h with a maximum amount after 5 h. At the same time, the percentage of inhibition was 54.1%, while PHT-SED was more effective than the standard drug since it inhibited edema in all time intervals. The percentage of inhibition in this case when the compound was administered at doses of 50 and 100 mg/kg was also higher and amounted to 65.09% and 81.08%, respectively. The authors chose the dose of 100 mg/kg to study the role of malondialdehyde (MDA) in preventing inflammatory edema. Injection of carrageenan caused a noticeable increase in this indicator (3.16 ± 0.19 nmol/mg of protein). In the case of prior administration of PHT-SED at a dose of 100 mg/kg or diclofenac at 25 mg/kg, MDA production was significantly reduced (1.32 ± 0.3 and 0.94 ± 0.2 nmol/mg protein, respectively) [175].

The anti-inflammatory effects of 6,6′-bieckol and 6,8′-bieckol from the same alga were studied by reproducing mouse ear edema under the action of xylene [176]. Both PTs suppressed ear edema at a dose of 75 nmol/mouse, with suppression efficiency of 61.8% and 55.7%, respectively, which was comparable to the effect of EGCG, epigallocatechin gallate, a substance with well-known anti-inflammatory properties [177]. Both PTs can be effective against acute inflammatory processes. Thus, brown algae PTs have anti-inflammatory and antioxidant effects, comparable to the effect of officinal preparations of this kind.

## 5. Oral Administration of Phlorotannins

Orally administered drugs are preferred over those issued parenterally. In this regard, the studies of Y. Sugiura et al., PTs’ anti-inflammatory and anti-allergenic effects are great interest. PTs (eckol, 6,6′-bieckol, 6,8′-bieckol, 8,8′-bieckol, florofucofuroeckols (PFF-A and PFF-B) from the brown alga *Eisenia arborea*, which were tested orally in mice with experimental ear edema. In addition, it was determined whether PTs suppressed the release of chemical mediators (histamine, leukotriene B4, and PGE2) from mast cells, as well as mRNA expression, activity COX-2, or both in the RBL-2H3 mast cell culture model [176].

Ear edema was induced in mice with three sensitizers: arachidonic acid (AA), 12-0-tetradecanoylphorbol-13-acetate (TPA), and oxazolone (OXA). In another study [12], the authors showed that the administration of phlorotannins directly suppressed ear edema by transdermal (applied to the skin?) administration of phlorotannins. The results of clinical trials with oral use of FT of the brown alga *A. nodosum* showed that phlorotannin metabolites were found in urine and blood plasma [18]. Based on these data, Sugiura et al. [176] suggested that phlorotannins from *E. arborea* also act systemically in ear edema. Orally administered terrestrial plant IFs are known to reduce IL-12 and IFNγ levels in OXA-induced mouse ear edema, and isoflavones suppress IFNγ and CCL24 mRNA expression and OXA-specific IgG levels in mice with DTH induced by OCA or 2,4-dinitrofluorobenzene. [178,179,180]. Based on the results obtained, it was concluded that the tested PT administered orally could modulate the inflammatory response [176].

All six PTs significantly inhibited the release of chemical mediators such as histamine, leukotriene B4, and prostaglandin E2 from mast cells. It is known that the expression and activity of COX-2 are of decisive importance in inflammation caused by the three sensitizers used in these experiments [160]. In the experiments of Sugiura et al. [88], at a concentration of 100 μM, all PTs suppressed the expression of COX-2 mRNA in RBL (mast cell) culture cells. Still, only 6,8′-bieckol was able to inhibit the expression of COX-2 at a concentration of 10 μM. Based on the literature data [103,181], the authors concluded that the inhibitory effects of the six purified FTs could be associated with COX-2 mRNA expression via MAPK/NF-kB signaling and inhibition of COX-2 activity in mast cells, which are abundant in inflamed tissues. FFB showed the best antiallergic effect and that its activity was higher than epigallocatechin gallate or tranilast.

## 6. Conclusions

Brown seaweed PTs have long been in the spotlight due to their numerous beneficial effects on the body, low toxicity, and excellent prospects for their use in medicine and the pharmaceutical and food industries [182]. However, a significant number of published studies on the anti-inflammatory and antioxidant effects of terrestrial and marine plant polyphenols substantiate the need for further in-depth research and the creation of low-toxic or non-toxic drugs, biologically active food supplements, and functional foods on their basis. Furthermore, the obtained results on anti-inflammatory activity substantiate the promising role of these compounds in the prevention and treatment of diseases with concomitant inflammatory processes, including malignant neoplasms, neurodegenerative diseases, obesity, type 2 diabetes, cardiovascular diseases, etc.

Currently, the process of accumulating and comprehending the results obtained is underway. Unfortunately, many problems are hindering the targeted clinical study of algal PTs and the creation of highly effective drugs. Most of the positive results have come from biochemical and cellular studies, with only a few results from in vivo laboratory animal experiments.

Preparation for the creation of dosage forms based on natural compounds requires obtaining structurally characterized compounds and comprehensive preclinical studies on various types of laboratory animals. In addition, it is necessary to create and clinically test effective agents that protect PT from biotransformation and degradation in the gastrointestinal tract to increase their therapeutic properties. Such studies are currently underway [182,183].

Using phlorotannins together with officinal drugs or other compounds from marine aquatic organisms in diseases with an inflammatory component is of great interest. In this regard, the study by Han et al. [184], in which the authors, with a positive result, used a mixture of PT and fucoidan, which have anti-inflammatory and antioxidant properties, to improve cognitive functions in mice with cognitive deficits induced by beta-amyloid peptide.

Clinical studies of the effectiveness of using these compounds in various pathological conditions as individual agents and adjuvant therapy have not been conducted to date. Considering all issues in a complex will provide an opportunity to determine whether algal phlorotannins can be viewed as an alternative therapeutic strategy to complement or replace existing traditional approaches to the treatment and prevention of diseases. Clinical studies on the bioavailability, efficacy, and safety of PTs will be required to determine the potential of these unique compounds for the prevention and treatment of disease in humans and animals.

In addition, the issue of the influence of limiting doses that have an anti-inflammatory effect requires careful study. An important point that requires detailed, including clinical research, is determining how the PTs of various algae affect the intestinal microbiota in inflammatory diseases of different origins. In addition, it is necessary to conduct further study into the mechanisms of action and the role of PTs in modulating numerous signaling pathways through which the anti-inflammatory effect of these biopolymers is carried out and the elimination of undesirable consequences of this complex process.

The promising results of clinical trials of phenols from terrestrial natural sources suggest the same or even more exciting result when using algal PTs. At the same time, taking into account modern approaches to the requirements of evidence-based medicine, clinical research should be comprehensive. The shift in focus from synthetically developed drugs to natural ones, particularly those derived from seaweeds, which have universal action and significant economic potential, opens up opportunities for obtaining innovative results in various fields—medicine, pharmacy, and the food industry.

## Figures and Tables

**Table 1:** Main targets and strategies of influence of phlorotannins on the activity of the transcription factor NF-kB during inflammation.

Main Targets and Strategies of Influence of Phlorotannins	References
Inactivation of LPS-induced transcriptional activity of NF-kB (dieckol *E. cava*).	[121,122]
Reduced expression of pro-inflammatory proteins: iNOS, pro-IL-Iβ and COX-2 (PT *F. vesiculosus*)	[85,123,124,125]
Inhibition of phosphorylation and degradation of protein- inhibitor IκBα (PT *F. vesiculosus*)	[126,127]
AKT/IkB-mediated inactivation signaling pathway NF-kB (dieckol *E. cava*)	[128]
Decreased production of pro-inflammatory cytokines TNFa and IL-6 (fucofuroeckol-A *Eisenia bicyclis*)	[129]
Reduction of overproduction of NO and PGP2 (trifuhalol A *Agarum cribrosum*)	[27]
Decreased activation of nuclear factor (NF-kB) and mitogen-activated proteinases (MAPKs)	[129,130]
Nrf2 and NF-kB interplay in cerebrovascular and neurodegenerative disorders: molecular mechanisms and possible therapeutic approaches.	[39,131,132]
Decreased expression of mRNA and proteins iNOS, COX-2 (PT *Ecklonia* sp. and *Eisenia* sp.)	[12,55]

**Table 2:** Main targets and strategies of influence of phlorotannins on the activity of the transcription factor MAPK during inflammation.

Main Targets and Strategies of Influence of Phlorotannins	References
Attenuation of MAPK signaling pathway activation due to the inhibition of p38 phosphorylation (trifuhalol, *Agarum cribrosum*).	[39]
Inhibited gene mRNA levels and protein expression of MMP-1, MMP-3 and MMP-13, iNOS and COX-2, and reduced inflammation via the MAPK signaling pathway (dieckol *E. cava*).	[137]
Inhibition of the MAPK signaling pathway has been confirmed by the use of MAPK inhibitors (UO126, SB203580, and SP600125) (eckol *E. cava*).	[39]
Inhibition of phosphorylation and degradation of protein-inhibitor IκBα (eckol *F. vesiculosus*).	[39]
